# The tumourigenicity of iPS cells and their differentiated derivates

**DOI:** 10.1111/jcmm.12062

**Published:** 2013-05-26

**Authors:** Zhiqiang Liu, Yu Tang, Shuanghong Lü, Jin Zhou, Zhiyan Du, Cuimi Duan, Zhiyan Li, Changyong Wang

**Affiliations:** a Department of Advanced Interdisciplinary Studies, Institute of Basic Medical Sciences and Tissue Engineering Research Center, Academy of Military Medical SciencesBeijing, China; b Department of Ultrasound, PLA 302 HospitalBeijing, China; c Laboratory of Oncology, Affiliated Hospital of Academy of Military Medical SciencesBeijing, China

**Keywords:** induced pluripotent stem cells, tumourigenicity, induced cardiac differentiation, bioluminescence imaging

## Abstract

Induced pluripotent stem cell (iPSC) provides a promising seeding cell for regenerative medicine. However, iPSC has the potential to form teratomas after transplantation. Therefore, it is necessary to evaluate the tumorigenic risks of iPSC and all its differentiated derivates prior to use in a clinical setting. Here, murine iPSCs were transduced with dual reporter gene consisting of monomeric red fluorescent protein (mRFP) and firefly luciferase (Fluc). Undifferentiated iPSCs, iPSC derivates from induced differentiation (iPSC-derivates), iPSC-derivated cardiomyocyte (iPSC-CMs) were subcutaneously injected into the back of nude mice. Non-invasive bioluminescence imaging (BLI) was longitudinally performed at day 1, 7, 14 and 28 after transplantation to track the survival and proliferation of transplanted cells. At day 28, mice were killed and grafts were explanted to detect teratoma formation. The results demonstrated that transplanted iPSCs, iPSC-derivates and iPSC-CMs survived in receipts. Both iPSCs and iPSC-derivates proliferated dramatically after transplantation, while only slight increase in BLI signals was observed in iPSC-CM transplanted mice. At day 28, teratomas were detected in both iPSCs and iPSC-derivates transplanted mice, but not in iPSC-CM transplanted ones. *In vitro* study showed the long-term existence of pluripotent cells during iPSC differentiation. Furthermore, when these cells were passaged in feeder layers as undifferentiated iPSCs, they would recover iPSC-like colonies, indicating the cause for differentiated iPSC's tumourigenicity. Our study indicates that exclusion of tumorigenic cells by screening in addition to lineage-specific differentiation is necessary prior to therapeutic use of iPSCs.

## Introduction

Induced pluripotent stem cells, embryonic stem cells (ESC)-like cells, can be generated from patients' own somatic cells with reprogramming, bypassing the ethical conflict and providing the possibility to resolve immune rejection associated with ESC application 1–3. Accordingly, it is supposed that the creation of iPSCs offers a renewed cell source and hope for cell-based therapy. Since 2006 when the iPSC was first established 4, the therapeutic potentials of iPSCs have been demonstrated in several disease models, including sickle cell anaemia, Parkinson's disease, myocardial infarction *etc*. 5–7. In these therapeutic applications, though encouraging results have been achieved, the teratoma formation after iPSC transplantation was also revealed by different groups. The tumorigenic risk, as confronted by ESC, is still a major hurdle preventing the further application of iPSCs, which should be carefully evaluated prior transplantation of iPSCs and iPSC-derivated cells.

One of the major applications for iPSC-therapy is myocardial repair 8, 9. Previously, several studies have evaluated tumorigenic risks of iPSCs during their cardiac application, but controversial results were observed 5–7. Nelson *et al*. reported myocardial repair using iPSCs in immunocompetent mice of MI without teratoma formation 7, while other reports found a high tumorigenesis after intramyocardial transplantation of iPSCs in mice and rats 10, 11, another study even observed more efficiently and faster teratoma formation by transplanted iPSCs than ESCs 12. Two possible reasons may be responsible for the fluctuant results. First, transplantation site-specific factors. Myocardium was a beating environment, mechanical compression and washout may lead to a great loss of cells from injected sites, that is, retained cells in transplantation sites varied from investigators and each operation, though the initially injected cells may be the same; Second, detection methods. With traditional histology, teratomas may be too small to be distinguished in gross examination and fail to be sampled for microscopic examination.

As for myocardial repair, there may be three applicable patterns for iPSCs: (*i*) transplantation of undifferentiated iPSCs 13, (*ii*) transplantation of predifferentiated iPSC (predifferentiated iPSC-derivates), (*iii*) transplantation of iPSC differentiated cardiac-lineage cells (iPSC-derived cardiac-specific cell type) 14. For safe application in future, a systemic evaluation for the tumourigenicity of the different iPSC-derivates is important, but one which is still devoid. In the study, different iPSC-derivates that may be used in myocardial repair were systemically evaluated for their tumourigenicity, including undifferentiated iPSCs, iPSC-derivates from induced cardiac differentiation (iPSC-derivates) and iPSC-derived cardiomyocytes (iPSC-CMs). In addition to traditional histology, BLI was performed for dynamic tracking the fate of transplanted cells, which ‘visualized’ the *in vivo* process of engraftment, proliferation and teratoma formation of different iPSC-derivates in a non-invasive and sensitive way.

## Materials and methods

### Culture and transduction of murine iPS cells

Induced pluripotent stem cell line (iPS-tet-B3) was a kind gift of Dr Gang Pei and Jiuhong Kang. The iPSC was generated from MEF of E13.5 129/C57 F1 mice embryos by transducing MEF with Yamanaka factors. The generation and characterization of iPSC see Ref. 15. Undifferentiated iPSCs were cultured on top of the mouse embryonic fibroblast feeder layers as explained in a previous report.

### Production of lentiviral vectors carrying tri-fusion reporter gene and establishment of iPS-TF line

The lentivirus vectors were produced by cotransfecting 293T cells with three plasmids, including reporter gene-carrying plasmid with fluc-mRFP-ttk fusion gene, packaging system ps PAX2 and envelop plasmid pMD2G, detailed protocol see Ref. 16. iPSCs were transduced at MOI of 15, successfully transduced cells were sorted by fluorescence-activated cell sorting (FACS; BD FACSVantage Diva, BD, Franklin Lakes, NJ, USA) based on the expression of mRFP.

### Embryoid formation and induced cardiac differentiation from iPS cells

Differentiation was initiated through embryoid body formation. Briefly, 1 × 10^6^ iPS cells in culture medium (with LIF) were transferred onto each petri dish (100 mm diameter). During suspension culture, EBs were formed and grew. At 5 day in suspension, EBs were transferred onto gelatin-coated culture dishes. Differentiation medium supplemented with 10^−3^ M vitamin C (vC) was used to induce iPSC-EBs into cardiomyocytes.

### Enrichment of cardiomyocytes

To obtain iPSC-CMs for transplantation studies, the contracting areas (14 days of vC-induced differentiation) were micro-dissected and dissociated as explained in a previous report 17.

### Cell transplantation

Nude mice were purchased from the Experimental Animal Center, Academy of Military Medical Science (Beijing, PRC). All experiments are approved by The Institutional Animal Care and Use Committee (IACUC) of the Chinese Academy of Military Medical Science, Beijing, China.

The iPSC-CMs were obtained from contracting EBs 14 days after vC-induced differentiation. 5 × 10^5^ iPSCs, iPSC-differentiated derivates (14 days of vC-induced differentiation), or iPSC-CMs were subcutaneously injected (in 10 μl PBS) into dorsal regions (one injection site in the upper region and two injection sites in the lower region) of nude mice (*n* = 6/group) as explained in a previous report 18.

### RNA extraction and reverse-transcription polymerase chain reaction

Total RNA was extracted with RNAprep pure Cell/Bacteria Kit (TIANGEN, Beijing, China) according to manufacturer's instruction. Reverse transcription reactions were performed using standard procedures to synthesize first-strand cDNA. The gene-specific primers were designed using primer3. The gene-specific primers used in PCR amplification are Nkx2.5 (5′-AGCAACTTCGTGAACTTTG-3′, 5′-CCGGTCCTAGTGTGGA-3′), Oct4 (5′-GGAGGAAGCCGACAACAATGAG-3′, 5′-TGGGGGCAGAGGAAAGGATACAG-3′), Sox2 (5′-CCAAGACGCTCATGAAGAACG-3′, 5′-GGAGTGGGAGGAAGAGGTAAC-3′), Nanog (5′-CTGGTCCCCACAGTTTGCCTA-3′, 5′-CTGGTCCCCACAGTTTGCCTA-3′), GAPDH (5′-AACGACCCCTTCATTGAC-3′, 5′-TCCACGACATACTCAGCAC-3′). Each PCR cycle consisted of denaturation at 94°C for 30 sec., and annealing/extension at 72°C for 45 sec. GAPDH was used as an internal standard.

### Bioluminescent imaging

After intraperitoneal injection of the reporter probe d-luciferin (150 mg/kg), mice were anaesthetized with inhaled isoflurane (2–3%) and imaged for 1–15 min. until the maximum signals were obtained. Bioluminescence signals were quantified in units of maximum photons/sec./cm^2^/steradian using living imaging software (Caliper Life Sciences, Hopkinton, MA, USA).

### Immunocytochemical staining

Cell samples were washed with PBS and then fixed with 4% paraformaldehyde. After permeabilization with 0.1% Triton X-100, the samples were incubated with the primary antibodies overnight at 4°C. The samples were washed with PBS to remove unconjugated primary antibodies. Fluorescein isothiocyanate-labelled secondary antibodies were added and incubated for 2 hrs at 37°C. For genomic DNA staining, samples were incubated with Hoechst33258 and observed under a fluorescent microscope (Olympus Optical, Melville, NY, USA). SOX2 and SSEA-1 were used to determine the undifferentiated state of iPSCs. The cTnT were used to determine differentiated cardiomyocytes.

### Histology and immunohistochemical staining

Animals were killed at 1 month after cell transplantation. Teratomas and surrounding tissues were sampled. Tissue samples were washed with PBS and fixed with 4% paraformaldehyde for 24 hrs. Then, the fixed tissue samples were embodied in paraffin. Four micrometre paraffin-embodied sections were prepared. Sections were stained with haematoxylin-eosin, or they were performed with immune-staining against pluripotent makers (OCT4, SOX2, NANOG and SSEA-1) as described above.

### Statistics

Data were expressed as mean ± SD and analysed with SPSS17.0. Student *t*-test was used for two groups' comparison. A value of *P* < 0.05 was considered statistically significant.

## Results

### Characterization and lentiviral transduction of iPSCs

**Fig. 1:**
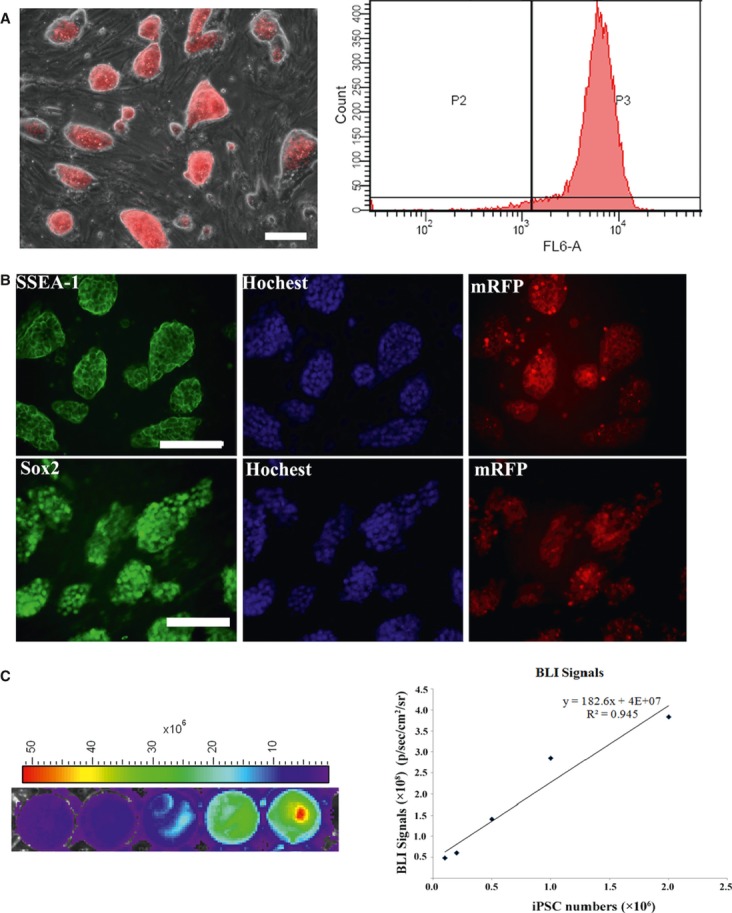
Culture and lentiviral transduction of induced pluripotent stem cells (iPSCs). (**A**) It could be observed under fluorescent microscope that transduced iPSCs strongly express monomeric red fluorescent protein (mRFP) in cytoplasm. Flowcytometry analysis indicates that more than 95% iPSCs are mRFP-positive after transduction and sorting; (**B**) immunostaining against SSEA-1 and Sox2 show that expression of reporter gene do not adversely affect iPSC pluripotency; (**C**) Bioluminescent imaging shows that BLI signals were linearly correlated with iPSC number (*R*^2^ = 0.95).

To track the survival, proliferation and teratoma formation in a non-invasive manner, iPSCs were transduced with dual reporter gene consisting of monomeric red fluorescent protein-firefly luciferase (mRFP-Fluc). Then, successfully transduced iPSCs were sorted by FACS based on the expression of mRFP. As shown in Figure 1, most sorted iPSCs (>95%) were mRFP-positive under fluorescent microscope as analysed by FACS. Immunostaining showed that transduced iPSCs maintained the expression of pluripotent markers SSEA-1 and Sox2 (Fig. 1B), suggesting that expression of reporter gene did not significantly influence iPSC pluripotency.

*In vitro* BLI was performed on different number of iPSCs to analyse fluc activity. As shown in Figure 1C, BLI signals were linearly correlated with the numbers of iPSCs (*R*^2^ = 0.945), indicating that fluc reporter and BLI technology could be used to quantitatively track iPSCs.

### Cardiac differentiation from iPSCs

**Fig. 2:**
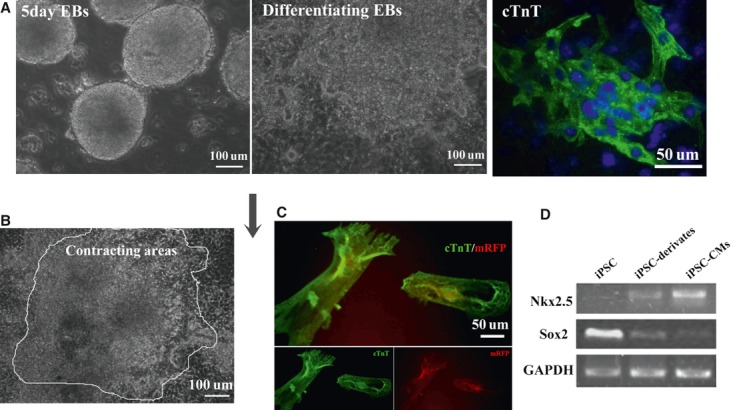
Differentiation and enrichment of induced pluripotent stem cell (iPSC)-CMs. Cardiac differentiation from iPSCs was induced with vitamin C (vC) through embryoid bodies (EB) formation. Five days EBs were seeded onto 0.1% gelatin-coated dishes and cultured with differentiation medium supplemented with 10^−4^ M vC. After 14 days differentiation, iPSC-derivates were collected and enriched. (**A**) EB formation, cardiac differentiation and characterization of differentiated cells; (**B**) Marked contracting areas for micro-dissecting; (**C**) immune-staining of micro-dissected and dissociated cardiomyocytes; (**D**) RT-PCR of iPSCs, iPSC-derivates and iPSC-CMs.

Cultivation of iPSCs in low-adherent dishes without LIF led to the formation of embryoid bodies (iPSC-EBs), Figure 2. When iPSC-EBs were transferred onto gelatin-coated dishes and vC was supplemented, EBs adhered and differentiated on the dishes. Within 1 week, contracting areas could be observed in differentiated EBs (Fig. 2). Compared with spontaneous differentiation (without vC), the ratio of contracting EBs was much higher in vC-induced differentiation. The size of contracting areas was also much larger (data not shown). Immunostaining demonstrated that contracting cells expressed cTnT, one of cardiac markers. The contracting EBs were micro-dissected and dissociated in to single cells, then the cells were reseeded onto 6-well plates for immunostaining. As shown in Figure 2, most of the sorted cells were cTnT positive. Counting under a fluorescent microscope showed that the percentage of cTnT-positive cells was >70%. Then, we tested the existence of pluripotent cells in different iPSC-derivates before transplantation with RT-PCR. The results showed that the expression of pluripotent genes, Sox2 iPSC-derivates from induced differentiation (by vC) was significantly lowered compared with undifferentiated iPSCs, while they were not detected in sorted iPSC-CMs.

### Non-invasive imaging in living mice

**Fig. 3:**
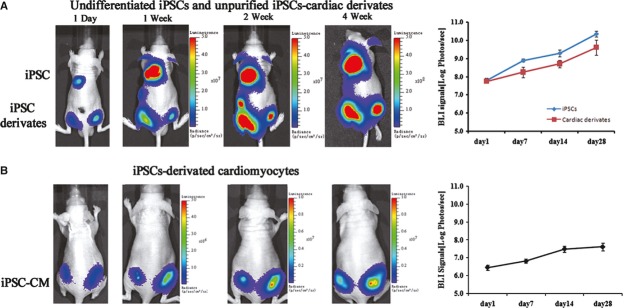
Tracking the engraftment and proliferation of transplanted cells with non-invasive bioluminescent imaging. (**A**) induced pluripotent stem cells (iPSCs) and iPSC-derivates proliferate dramatically with time after transplantation, indicating the teratoma formation; (**B**) Imaging of iPSC-CMs after transplantation.

After subcutaneous injection in nude mice, non-invasive imaging showed that BLI signals increased rapidly with time in animals receiving undifferentiated iPSC injection (Fig. 3A), indicating that undifferentiated iPSCs proliferated dramatically in transplanted sites. BLI signals in animals receiving injection of iPSC-differentiated derivates showed a similar tendency to that of undifferentiated iPSCs. As shown in Figure 3A, within 1 week after transplantation, BLI in iPSC-derivate transplanted animals increased much slower than that in iPSC-transplanted animals, however, they increased at a similar rate after 1 week. These results indicated that there may be some pluripotent cells in iPSC-derivates and they would recover undifferentiated iPSC properties with time after transplantation, thus demonstrating similar proliferating capacity as undifferentiated iPSCs.

Different from the transplantation of undifferentiated iPSCs and iPSC-derivates, transplantation of iPSC-CMs only resulted in a slight increase in BLI signals at a time (Fig. 3B). As shown in Figure 3B, the BLI signals from animals receiving iPSC-CMs injection increased within 2 weeks after transplantation, and then the signal curve entered a plateau phase. The signals at 2 weeks were comparable to those at 4 weeks. The results indicated that iPSC-CMs possessed proliferative capacities, this is consistent with previous reports about iPSC or ESC differentiated cardiomyocytes. However, the proliferation of iPSC-CMs could be maintained only for a short time.

### Tumorigenic capacities of iPSCs and their derivates

**Fig. 4:**
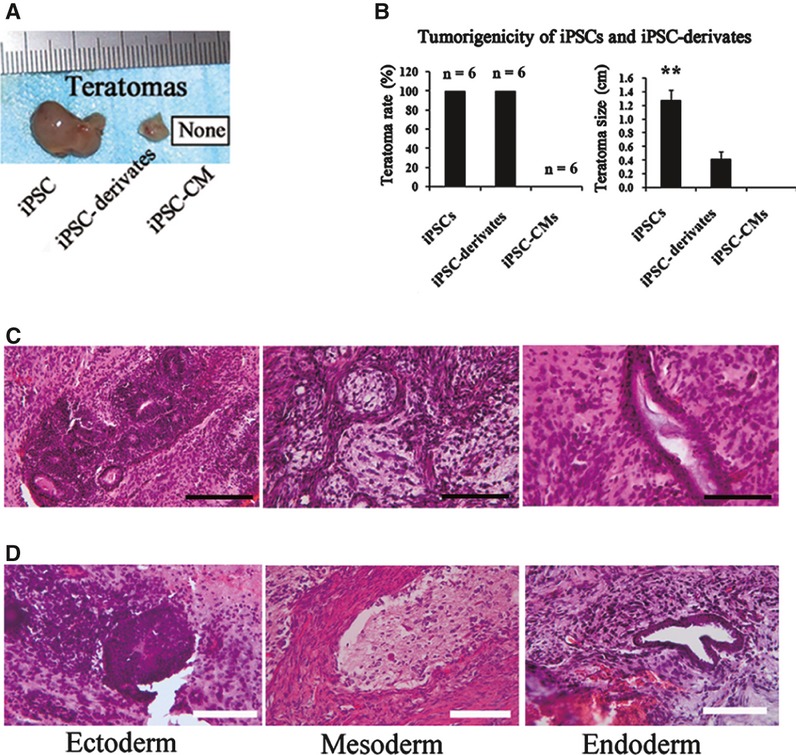
Teratomas formed by undifferentiated induced pluripotent stem cells (iPSCs) and iPSC-derivates. (**A**) visible teratomas formed by iPSCs and iPSC-derivates; (**B**) comparison of tumorigenic rate and teratoma size produced by different iPSC-derivates; (**C**) different structures in teratomas formed by iPSCs and (**D**) different structures in teratomas formed by iPSC-derivates. **P < 0.01 compared with iPSC-derivates.

Within 2 weeks, visible tumours from undifferentiated iPSCs could be observed on the back of mice. After animals were killed at 4 weeks, tumours were isolated and compared, both iPSCs and iPSC-derivates formed visible tumours in all injection sites (6/6), while no visible tumours were observed in iPSC-CM injected sites (0/6), as shown in Figure 4A and B. iPSC-formed tumours, which were much larger that iPSC-derivate formed ones (*P* < 0.01). From haematoxylin and eosin staining sections, typical structures of tri-germ layers were observed both in iPSC and iPSC-derivate formed teratomas (Fig. 4C and D). Still, no teratoma structure and tumorigenic cells were detected in iPSC-CMs transplantation samples.

### Long-term residual of tumorigenic cells during directional differentiation of iPSCs

**Fig. 5:**
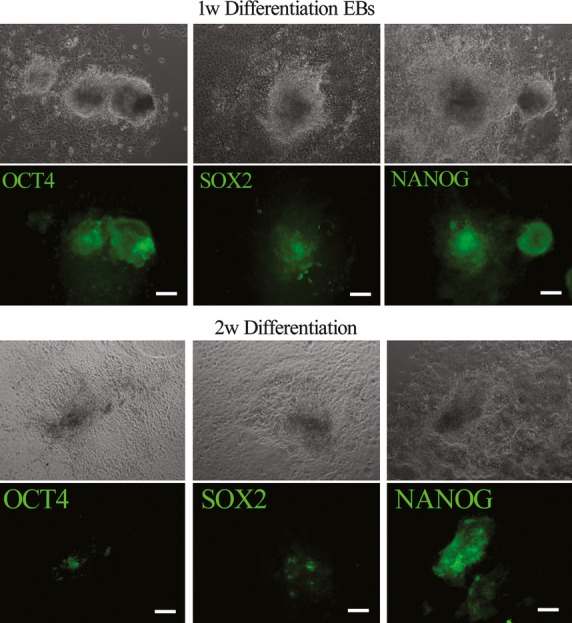
Persistent existence of pluripotent cells along induced pluripotent stem cell (iPSC) differentiation. Pluripotent cells positive for OCT4, SOX2 and NANOG remain in differentiated embryoid bodies (EBs) after 2 weeks of induced differentiation, though the ratio of pluripotent cells decrease with differentiation (bar = 100 μm).

**Fig. 6:**
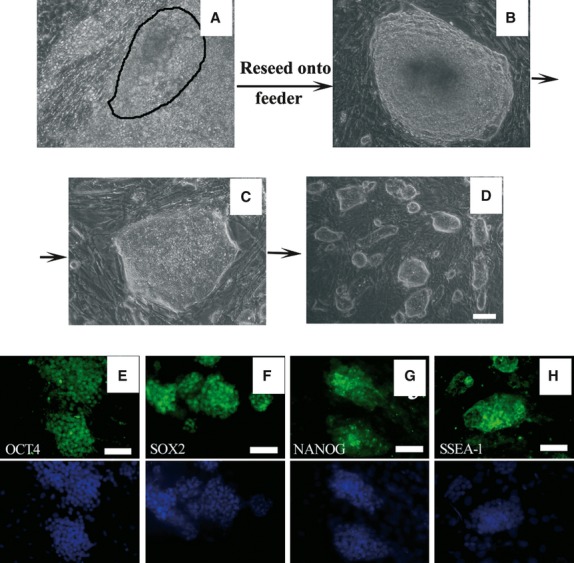
Redifferentiation of induced pluripotent stem cell (iPSC)-derivates on feeder layers. (**A**) Differentiated iPSC-embryoid bodies (EBs) induced by vitamin C (vC) for 14 days; (**B**–**D**) When reseeded onto feeder layers, differentiated iPSC-derivates dedifferentiated and restored to iPSC state with passaging, (B) passage1, (C) passage2, (D) passage3; (**E**–**F**) recovered iPSCs express pluripotent markers like undifferentiated iPSCs.

To determine the long-term existence of tumorigenic cells during the directional differentiation of iPSCs, immunostaining against pluripotent markers, Oct4, Sox2 and Nanog was performed on differentiating iPSC-EBs (induced with vC). The results showed that pluripotent cells positive for these markers persistently existed during iPSC-EB differentiation, though these cells were decreasing with time as observed in Figure 5. Even after 1 month differentiation, these pluripotent cells could be still observed (data not shown). In transplantation experiment, we found that some cells in iPSC-derivates may revert to undifferentiated iPSC state and contribute to teratoma formation *in vivo*. To test the presumption, some cell clusters from 1 month differentiated derivates (5 days EBs and 30 days differentiation) were dissected and reseeded onto feeder layers. When the cells were cultured as undifferentiated iPSCs, some iPSC-like colonies were recovered (Fig. 6A–C). After two passages on feeder layers, these colonies were nearly the same as undifferentiated iPSC colonies, we call them Re-iPSC (recovered iPSCs, Fig. 6D). Furthermore, immunostaining demonstrated that the re-iPSC possessed a similar phenotype as undifferentiated iPSCs, expressing pluripotent markers Oct4, Sox2 and Nanog (Fig. 6E–H).

**Fig. 7:**
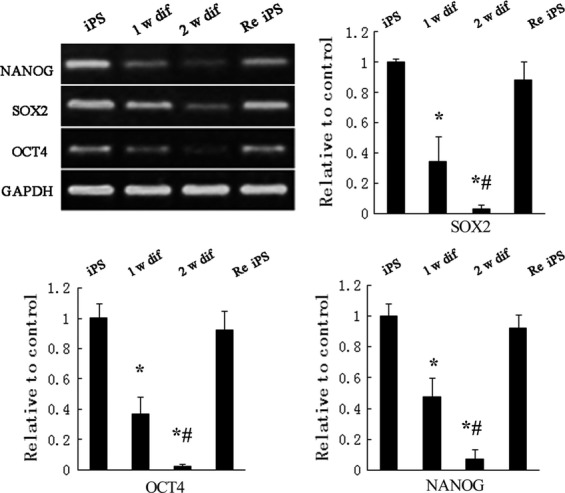
Pluripotent gene expression in induced pluripotent stem cells (iPSCs) during induced differentiation and dedifferentiation. The expression of pluripotent genes decrease with differentiation of iPSC-embryoid bodies (EBs), but they were rapidly recovered to the level comparable to undifferentiated iPSCs (As shown Re-iPSCs). Re-iPS indicated the iPSCs recovered from 2 week-differentiated cells, which were reseeded onto feeder layers. *P < 0.01 compared with iPSCs, #*P* < 0.01 compared with 1 week differentiation.

To test the pluripotent gene expression during iPSC differentiation and in Re-iPSCs, RT-PCR analysis was performed (Fig. 7). Consistent with immunostaining results, the expression of pluripotent genes (including Oct4, Sox2, Nanog) was decreasing with iPSC differentiation. After 2 weeks differentiation, the expression of pluripotent genes was reduced more than 10 times compared with undifferentiated iPSCs (*P* < 0.01). However, the expression of pluripotent genes in Re-iPSC was nearly the same levels as undifferentiated iPSCs. It should be noticed that these Re-iPSC were obtained from 1 month differentiated derivates, which were passaged only for two times on feeder layers. The results suggested that the pluripotent genes in iPSC-derivates (even after long-term differentiation) could be rapidly recovered under suitable conditions.

## Discussion

It was supposed that iPSC possessed a promising perspective for clinical application because of its therapeutic advantages, such as high pluripotency, autologous availability without ethical issues, *etc*. 19, 20. However, as an ESC-like cell type,its tumourigenicity has become a main concern 10, 21. It has been found that incompletely differentiated cells existed both in ESC and iPSC spontaneous differentiation, making differentiated derivates tumorigenic 22, 23. In the study, we further evaluated the tumorigenicities of different iPSC-derivates systemically. Consistent with previous reports, we found with *in vitro* experiment, the long-term existence of incompletely differentiated cells during induced iPSC differentiation, and these cells could dedifferentiate and restore their undifferentiated state. Furthermore, there were two novel findings in the studies: (1) we firstly ‘visualized’ the dynamic process that iPSC-derivates dedifferentiated and formed teratomas *in vivo* using BLI and (2) confirmed that physical micro-dissection could efficiently eliminate tumorigenic cells from differentiated iPSC-derivates, providing a possible way for the safe use of iPSCs.

In previous studies, fluctuant results were observed about the tumourigenicity of iPSCs 7, 11, 12, 21. We supposed that transplantation sites (*e.g*. beating myocardium as mentioned in the introduction) and detection methods (histological methods as mentioned in the introduction) may be important factors that were responsible for the controversy about the iPSC tumourigenicity. In the study, we adopted different strategies to minimize the variance produced by the above-mentioned uncertain factors. To minimize the cell loss because of transplantation environment, subcutaneous regions of nude mice were selected instead of beating myocardium for transplantation; furthermore, BLI was used for graft detection besides histological detection. The method was sensitive, non-invasive and could be used for longitudinal tracking of grafts 24–26. Based on BLI, we could obtain visualized information about graft localization and quantity, avoiding the bias in cell transplantation and detection.

It has been reported that residual of incompletely differentiated cells was the major reason for tumourigenicity of ES/iPSC-derivated cells 22, 23. In the study, we demonstrated the *in vivo* dynamic process that derivates from induced iPSC differentiation proliferated, dedifferentiated and formed teratomas. As shown by BLI, iPSC-derivates proliferated more slowly than iPSCs (smaller slope in BLI curve) early after transplantation (the first week), but the proliferation of iPSC-derivates was increasingly faster with time (increasing slope in BLI curve). After 2 weeks, they had a similar proliferation rate, indicating that injected iPSC-derivates underwent certain redifferentiation, which resulted in a higher proliferation capacity. At 4 weeks, both iPSCs and iPSC-derivates formed teratomas. It is noteworthy that the initially transplanted cells were much different in their pluripotency. However, we observed that teratomas from iPSCs and iPSC-derivates were much similar in structure and components, further suggesting that some cells in iPSC-derivates redifferentiated *in vivo* and restored their high pluripotency like iPSCs.

Although all animals receiving iPSCs and iPSC-derivates formed visible teratomas, teratomas formed by iPSC-derivates were significantly smaller than those formed by iPSCs, indicating that the tumourigenicity of iPSCs was depressed to some extent by induced differentiation, or some cells derivated from iPSC differentiation have lost tumourigenicity. That is, there could be three cell types in iPSC-derivates considering their tumorigenicities: (1) cell population whose tumourigenicity was similar to iPSCs. These cells may undergo certain differentiation, but they could dedifferentiate and restore to iPSC state rapidly under suitable condition; (2) cell populations whose tumorigenicities were depressed. It may need more cell quantities and longer time for the cells to form teratomas because of their lower tumorigenic capacity; (3) cell populations that have lost the tumorigenic capacity. These cells may be safe after transplantation, which would be important seeding cells in cell therapy. Therefore, we further investigated tumourigenicity of specific cell types from induced iPSC differentiation by removing other cell types. In the study, iPSC-CMs were investigated considering their potential application in myocardial regeneration.

Actually, there have been several methods for cardiomyocyte selection, including immune methods, genetic methods, flow cytometry based on the cardiac markers, or selection based on dye staining (*e.g*. TMRM) 27–30. In the study, we selected iPSC-cardiomyocytes using a simple micro-dissection of contracting areas of differentiated EBs 17. The method is convenient and practical, however, a drawback is that the selection efficiency is not high enough 31. We obtained about 70–80% iPSC-cardiomyocytes by the method. However, we found that the method is practical to remove the tumorigenic cells from induced iPSC-derivates, no a signal teratoma was observed in the study as determined by BLI and histology. Although iPSC-CMs also underwent proliferation early after transplantation, the proliferation was very limited and could not involve the dedifferentiation. It should mainly be resulted from the proliferating nature of stem cell-derivated cardiomyocytes; this has been confirmed previously by different reports 29.

The iPSC line used in the study was derivated by reprogramming fibroblasts with Yamanaka's factors. The reprogramming method is based on integrating viral vectors, which integrate randomly into the host genome. Therefore, risks may still exist for future reprogramming in some circumstances. At present, several other safer methods with non-integrating vectors have been developed, such as recombinant proteins (DNA-free strategy) 32, miRNA strategy 33, *etc*. The iPSC lines derived from these methods would be more therapeutically applicable; the tumourigenicity of such iPSC lines and their derivates deserves further investigation.
